# Molecular and Cellular Mechanisms of Myelodysplastic Syndrome: Implications on Targeted Therapy

**DOI:** 10.3390/ijms17040440

**Published:** 2016-03-24

**Authors:** Harinder Gill, Anskar Y. H. Leung, Yok-Lam Kwong

**Affiliations:** Department of Medicine, Queen Mary Hospital, Hong Kong, China; gillhsh@hku.hk (H.G.); ayhleung@hku.hk (A.Y.H.L.)

**Keywords:** myelodysplastic syndrome, gene mutations, prognostication, target therapy

## Abstract

Myelodysplastic syndrome (MDS) is a group of heterogeneous clonal hematopoietic stem cell disorders characterized by cytopenia, ineffective hematopoiesis, and progression to secondary acute myeloid leukemia in high-risk cases. Conventional prognostication relies on clinicopathological parameters supplemented by cytogenetic information. However, recent studies have shown that genetic aberrations also have critical impacts on treatment outcome. Moreover, these genetic alterations may themselves be a target for treatment. The mutation landscape in MDS is shaped by gene aberrations involved in DNA methylation (*TET2*, *DNMT3A*, *IDH1/2*), histone modification (*ASXL1*, *EZH2*), the RNA splicing machinery (*SF3B1*, *SRSF2*, *ZRSR2*, *U2AF1/2*), transcription (*RUNX1*, *TP53*, *BCOR*, *PHF6*, *NCOR*, *CEBPA*, *GATA2*), tyrosine kinase receptor signaling (*JAK2*, *MPL*, *FLT3*, *GNAS*, *KIT*), RAS pathways (*KRAS*, *NRAS*, *CBL*, *NF1*, *PTPN11*), DNA repair (*ATM*, *BRCC3*, *DLRE1C*, *FANCL*), and cohesion complexes (*STAG2*, *CTCF*, *SMC1A*, *RAD21*). A detailed understanding of the pathogenetic mechanisms leading to transformation is critical for designing single-agent or combinatorial approaches in target therapy of MDS.

## 1. Introduction

Myelodysplastic syndrome (MDS) is a group of clonal hematopoietic stem cell disorders characterized by ineffective hematopoiesis leading to cytopenia, and a significant risk of evolution to acute myeloid leukemia (AML) [1]. Conventional prognostic scoring of MDS is based on the extent of cytopenia, the percentage of bone marrow blast infiltration, and karyotypic abnormalities [2,3]. Risk categories based on prognostic scoring determine the therapeutic approaches. Treatment of high-risk MDS involves the use of hypomethylating agents (HMA) [4,5,6,7,8] and allogeneic hematopoietic stem cell transplantation (HSCT) in younger patients [1]. Clinical studies with HMAs including azacitidine and decitabine have shown a response rate of about 40% in high-risk patients and a median response duration of merely 9 to 15 months. HMA failure is associated with a poor prognosis and a median survival of less than 5 months [9,10]. Allogeneic HSCT is potentially curative for 25%–60% of patients depending on the risk category, but is restricted by age and donor availability. Treatment failure following HMA and allogeneic HSCT in high-risk MDS remains an unmet clinical need.

**Figure 1:**
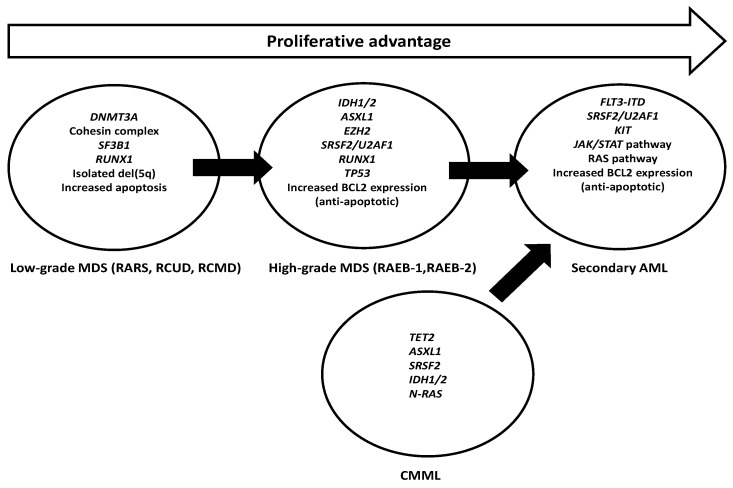
Gene mutations and their roles in disease progression in myelodysplastic syndrome (DNMT3A: DNA methyltransferase 3A; SF3B1: splicing factor 3b subunit 1; RUNX1: runt related transcription factor 1; IDH1/2: isocitrate dehydrogenase 1/2; ASXL1: additional sex combs like 1; EZH2: enhancer of zeste 2 polycomb repressive complex 2 subunit; SRSF2: serine/arginine-rich splicing factor 2; U2AF1: U2 small nuclear RNA auxiliary factor 1; TP53: tumor protein p53; BCL2: B-cell CLL/lymphoma 2; FLT3-ITD: fms-like tyrosine kinase-3 internal tandem duplication; KIT: KIT proto-oncogene receptor tyrosine kinase; JAK/STAT: Janus kinase/signal transducer and activator of transcription; RAS: rat sarcoma viral oncogene homolog; TET2: ten-eleven translocation methylcytosine dioxygenase 2; N-RAS: neuroblastoma RAS oncogene).

The pathophysiology of MDS and its progression to AML involve cytogenetic, genetic, and epigenetic aberrations [1] (Figure 1). Genome-wide and targeted analyses from next-generation sequencing have identified novel mutations of prognostic and therapeutic significance [1,11,12,13]. Recurrent mutations in more than 45 genes are found in over 85% of cases [1,14]. These mutations are found in genes regulating DNA methylation (*DNMT3A*, *TET2*, *IDH1/2*), post-translational chromatin modification (*EZH2*, *ASXL1*), transcription regulation (*TP53*, *RUNX1*, *GATA2*), the RNA spliceosome machinery (*SF3B1*, *U2AF1*, *SRSF2*, *ZRSR2*), cohesion complexes (*STAG2*), and signal transduction (*JAK2*, *KRAS*, *CBL*) [14]. Mutations in *TP53*, *EZH2*, *ETV6*, *RUNX1*, *SRSF2* and *ASXL1* portend inferior survivals [14]. These mutations may also predict responses to HMA and allogeneic HSCT. Furthermore, specific mutations, such as internal tandem duplication of *FLT3* (*FLT3*-ITD), have been observed during disease progression and are potential therapeutic targets [15,16]. Therefore, a better understanding of the molecular landscape in MDS has important implications on treatment response, prognostication, and novel molecular therapeutic targeting.

## 2. Targeting Genes Involved in DNA Methylation

Aberrant DNA methylation represents one of the important mechanisms underlying altered epigenetic regulation in MDS [11]. Frequently mutated genes relevant to this pathway include *TET2*, *DNMT3A*, and *IDH1/2* [1,11].

### 2.1. TET2 Mutations

The TET family comprises the dioxygenase proteins TET1, TET2, and TET3. TET2, its gene mapping to chromosome 4q24, modulates hydroxymethylation by catalyzing the conversion of 5-methylcytosine to 5-hydroxymethylcytosine, which is an intermediate of DNA methylation that blocks the building of silencing proteins to methylated DNA [17]. *TET2* mutations are observed in a range of myeloid neoplasms including AML, MDS, and myeloproliferative neoplasm (MPN) [18,19]. In MDS, *TET2* mutations are found in 20%–25% of patients, but occur at a higher frequency of 30%–60% in chronic myelomonocytic leukemia (CMML) [20,21,22,23]. The prognostic impact of *TET2* mutations on survivals in MDS is controversial. Large cohort studies showed that *TET2* mutations did not appear to impact on overall survivals (OS) [24,25]. On the other hand, *TET2* mutations have also been shown to confer superior survivals [23]. Furthermore, *TET2* mutations may predict a more favorable response to HMAs in high-risk patients [1,11,19].

### 2.2. DNMT3A Mutations

The DNMT family comprises the enzymes DNMT1, DNMT3A, and DNMT3B, and catalyzes the transfer of methyl group on the 5′-position of cytosines located at CpG dinucleotides [26]. They are involved in physiological processes that include imprinting, X-chromosome inactivation, differentiation, proliferation, and apoptosis [26]. DNMT1 maintains DNA methylation during DNA replication, while *DNMT3A* and *DNMT3B* catalyze DNA methylation. *DNMT3A* is highly expressed in T lymphocytes and neutrophils, while *DNMT3B* is downregulated in hematopoietic differentiation. Aberrant CpG island promotor methylation in tumor suppressor genes is an important pathogenetic mechanism in malignant tumors, suggesting that DNMTs play important roles in oncogenesis.

*DNMT3A* mutations occur in 30%–35% of AML with normal karyotype, and about 10% of MDS and 20% of T-lineage acute lymphoblastic leukemia [27,28,29]. *DNMT3A* mutations result in loss of function, and are present in pre-leukemic hematopoietic stem cells, remaining stable through disease evolution to MDS and AML [30,31]. MDS patients with *DNMT3A* mutations have shorter OS and higher risks of leukemic transformation [32,33]. *DNMT3A* mutations are specific biomarkers of positive response to DNA methyltransferase inhibitors [34]. Azacitidine and decitabine incorporate into DNA, resulting in proteosomal degradation of DNMTs. Guadecitibine (SGI-110) is a second generation HMA and a dinucleotide of decitabine and deoxyguanosine [28], with its use leading to extended decitabine exposure via resistance to deamination. Apart from DNA methylation, histone deacetylation is the other post-translational modification involved in the silencing of genes. Histone deacetylases (HDAC) are enzymes catalyzing histone acetylation, which are over-expressed in various malignancies including AML. However, HDAC inhibitors have limited efficacy as single agents. Phase II trials of HDAC inhibitors in combination with HMAs are ongoing. Examples of HDAC inhibitors that have shown activity in combination with HMAs include pracinostat, vorinostat, and valproic acid [28].

### 2.3. Isocitrate Dehydrogenases 1 and 2 (IDH1 and IDH2) Mutations

Isocitrate dehydrogenases 1 and 2 (IDH1 and IDH2) are a family of NADP-dependent enzymes critically involved in the conversion of isocitrate to α-ketoglutarate (α-KG) [27,28]. *IDH1/2* mutations are heterozygous and occur mostly at residues R132 in *IDH1*, and R140 or R172 in *IDH2*. *IDH1* and *IDH2* mutations are mutually exclusive with *TET2* mutations in AML, suggesting that they play a similar mechanistic role. Mutant IDH1 and IDH2 proteins have a reduced affinity for isocitrate, but acquire a neomorphic function, not shared by the wild-type enzymes, in converting α-KG to 2-hydroxyglutarate (2-HG) via oxidation of NADP and release of carbon dioxide. As a result, abnormal intracellular accumulation of 2-HG inhibits the dioxygenase enzymes JumonjiC (JmjC) and TET2, prolyl/lysyl hydroxylases, and cytochrome C oxidase (COX). These enzyme inhibitions lead to epigenetic dysregulations [27,28] postulated to be involved in oncogenesis, so that 2-HG is considered an oncometabolite. *IDH1/2* mutations are seen in patients with cytogenetically normal AML, MDS, MPN, angioimmunoblastic T-cell lymphoma, glioma, cholangiocarcinoma, and chondrosarcoma [27,35]. In leukemogenesis, additional cooperative genetic mutations (such as in *HOXA9* and *NPM1*) are required. *IDH1/2* mutations are seen in 2%–12% of MDS [36,37], being more prevalent in refractory anemia with excess blasts-2 (RAEB-2) than other low-grade MDS [38]. *IDH1* mutations, particularly those at codon 132, are associated with inferior OS [36]. *IDH2* mutations in MDS are frequently present concurrently with *DNMT3A*, *ASXL1*, and *SRSF2* mutations and also predict inferior OS, especially those at codon 172 [37]. *IDH1/2*-mutated myeloid malignancies are potentially targetable by direct blockade of the mutant enzymatic activity or the associated metabolic pathways [28]. The IDH1-R132H inhibitor AGI-5198 is active against glioma cells *in vitro* [39]. The IDH2-R140Q inhibitor AGI-6780 induces changes in DNA methylation and histone states, resulting in differentiation of AML cell lines and primary AML cells [40,41]. The IDH1 inhibitor HMS-101 blocks colony formation of primary *IDH1*-mutated AML. In a phase I trial, the oral IDH2 inhibitor AG-221 induced an overall response rate of 62.5% and a complete response rate of 37.5% in patients with relapsed or refractory *IDH2*-mutated AML and MDS [27]. AG-221 achieved a durable inhibition of plasma 2-HG in more than 90% of patients with *IDH2*-R140Q mutation and 50% of patients with *IDH2*-R172K mutation [28]. The selective oral IDH1 inhibitor AG-120 decreased intracellular 2-HG levels and resulted in growth inhibition and differentiation in primary *IDH1*-mutant AML cells *ex vivo*. A phase I study evaluating AG-120 is ongoing. Intravenous asparaginase *Erwinia chrysanthemi* (Erwinase) is currently being evaluated in *IDH1*-mutated adult AML [28]. Targeting of the altered metabolic pathways in *IDH1/2*-mutated AML is another approach, and drugs tested include glutaminidase inhibitors (BPTES, zaprinast and CB-839) and NADPH inhibitors (epigallocatechin-3-gallate) [27].

## 3. Targeting Mutation in Histone Modification Genes

### 3.1. EZH2 Mutations

EZH2, a histone-lysine *N*-methyltransferase enzyme, catalyzes the addition of methyl groups to histone H3 at lysine 27 (H3K27). It is a key component of Polycomb repressive complexes (PRCs), participating in transcription repression [28]. *EZH2* mutations can be detected in lymphomas and myeloid malignancies. In MDS, *EZH2* mutations are seen in 6%–12% of patients. *EZH2* mutations frequently co-exist with *TET2* mutations, and are associated with disease transformation. Consequently, *EZH2* mutations are observed in high-grade MDS, and are associated with a higher risk of secondary AML and worse OS. A specific EZH2 inhibitor, GSK-126, has been shown to be active in *EZH2*-mutated lymphomas [28], and has yet to be tested in myeloid malignancies.

### 3.2. ASXL1 Mutations

The ASXL1 protein, its gene mapping to chromosome 20q11.21, regulates histone modification by interacting with the PRC2 [11]. PRC2 methylates H3K27 and is a key regulator of hematopoiesis [42,43]. Biologically, *ASXL1* loss is associated with hematopoietic transformation and increased self-renewal [28]. *ASXL1* mutations are found in 11% of patients with MDS, portending an inferior OS, and 43% of patients with CMML, being associated with a higher risk of secondary AML [44,45].

### 3.3. UTX Mutations

The *UTX* gene or the lysine (K)-specific demethylase 6A (*KDM6A*) gene is a member of the JumonjiC family and encodes an H3K27 demethylase [46,47]. UTX regulates histone methylation in conjunction with other epigenetic modifiers. *UTX* mutations are seen in 8% of patients with CMML and 1% of patients with MDS [11]. The prognostic impact of *UTX* mutation is unclear. *UTX* (*KDM6A*) can be targeted by small molecule inhibitors that interfere with its activity [28]. GSKJ1 and GSKJ4 are two new agents developed with inhibitory effect against KDM6A activity [28].

## 4. Targeting Mutations in the RNA Splicing Genes

RNA splicing is a process where mature RNA is formed from pre-messenger RNA (pre-mRNA) through intron removal and exon splicing. Mutation of genes involved in RNA splicing, including *SF3B1*, *SRSF2*, *ZRSR2*, and *U2AF1/2*, occur in approximately 50% of MDS patients [11].

### 4.1. SF3B1 Mutations

*SF3B1*, encoding the subunit 1 of the splicing factor 3b complex, is an essential component of the U2 snRNP that recognizes the 3′ splice site at intron-exon junctions [48]. *SF3B1* mutations are seen in 57%–75% of patients with refractory anemia with ring sideroblasts (RARS), and 6%–18% of patients with other subtypes of MDS [49,50]. Mutant SF3B1 downregulates genes essential in the mitochondrial pathways. These include the *ACACA* (acetyl-coenzyme A carboxylase alpha) and the *RGL1* (ral guanine nucleotide dissociation stimulator like-1) genes. *SF3B1*-mutated RARS have abnormal splicing of the *ABCB7* gene in the mitochondria, which leads to deficiency of the ABCB7 protein, resulting in mitochondrial iron overload, reduced heme synthesis, and ineffective erythropoiesis [11,19]. *SF3B1*-mutated MDS is associated with thrombocytosis, increased ring sideroblasts, fewer cytopenias, and lower blasts percentage [50]. *SF3B1* mutations are associated with a favorable prognosis and prolonged survivals [50].

### 4.2. SRSF2 Mutations

*SRSF2*, encoding the serine/arginine-rich splicing factor 2, is critically involved in splice site selection, spliceosome assembly, and constitutive and alternative splicing [51]. *SRSF2* mutations are stable during disease evolution in MDS, suggesting that they may play a role in disease initiation [51]. *SRSF2* mutations are seen in 11%–15% of patients with MDS, frequently co-existing with *RUNX1*, *IDH1*, *IDH2*, and *ASXL1* mutations [11], and confer an inferior OS [52,53].

### 4.3. ZRSR2 Mutations

ZRSR2 encodes a subunit of U2 auxiliary factor. *ZRSR2* mutations occur in 3% of patients with MDS. Their prognostic significance is unclear [54].

### 4.4. U2AF1 Mutations

*U2AF1* gene, mapping to chromosome 21q22.2, encodes the U2 auxiliary factor that facilitates the binding of U2 snRNP to the pre-mRNA branch site [54]. Recurrent mutations of the *U2AF1* gene occur in 9% of patients with MDS [54]. The prognostic impact of *U2AF1* mutations remains unclear [11,54].

## 5. Targeting Mutations in Transcription Factor Genes

### 5.1. RUNX1 Mutations

RUNX1 is a key regulator of myeloid differentiation, and was first described in a familial platelet disorder associated with leukemic evolution [55]. *RUNX1* mutations are found in 24% of patients with RAEB-1/RAEB-2 and secondary AML [56,57], and independently confer an inferior prognosis [11].

### 5.2. BCOR/BCORL1 Mutations

BCOR and BCORL1 function as co-repressors. They co-repress BCL6 function and play key roles in embryonic development [58,59,60]. In addition to its role in transcription regulation, BCOR/BCORL1 are also a component of PRCs [60]. *BCOR* and *BCORL1* mutations are seen in approximately 5% and 1% of patients with MDS and are associated with inferior OS [61].

### 5.3. ETV6 Mutations

The ETS is a family of transcription factors that play important roles in hematological malignancies. ETV6 (ets-variant 6) co-represses transcription of ETS. ETV6 gene aberrations are commonly secondary to chromosomal translocations. The most common chromosomal translocation involving ETV6 is t(3;12)(q26;p13) [11]. In addition, deletions or somatic mutations of *ETV6* have been described in patients with MDS and AML, and are associated with monosomy 7 [11]. *ETV6* rearrangements are seen in about 1% of MDS and confer a poor prognosis in MDS [62].

### 5.4. SETBP1 Mutations

SETBP1 is a nuclear protein that binds to SET, interacting with PP2A, a tumor suppressor gene [11]. It plays an important role in the regulation of cellular proliferation. *SETBP1* mutations are seen in 2%–4% of MDS, 6%–15% of CMML, and 24%–32% of atypical chronic myeloid leukemia [11]. Other associations with *SETBP1* mutations include leucocytosis, monosomy 7, 7q deletion, isochromosome 17p, and mutations of the other epigenetic regulators *ASXL1*, *EZH2*, and *SRSF2* [63,64]. *SETBP1* mutations are associated with inferior survivals and leukemic transformation [63,64].

### 5.5. GATA2 Mutations

GATA2 is a transcription factor critical in hematopoietic stem cell proliferation and survival. *GATA2* mutations have been described in familial MDS and may play a role in evolution to MDS and AML in patients with chronic neutropenia [11].

## 6. Targeting DNA Repair/Tumor Suppressor Genes

### TP53 Mutations

*TP53* is a tumor suppressor gene with key functions in regulating cell cycle and DNA repair [65,66]. *TP53* mutations are detected in about 9% of MDS, with a higher frequency in cases of with isolated 5q deletion and complex karyotypes [67]. *TP53* mutations are associated with a high risk of leukemic transformation and a poor OS [11]. Therapeutic targeting of TP53 has been explored in MDS patients with del(5q). Canersen is an antisense oligonucleotide complementary to TP53, which is capable of suppressing mutant p53 expression and restoring impaired erythropoiesis in patients with del(5q) not responding to lenalidomide therapy [68].

## 7. Targeting Mutations in Signal Transduction

### 7.1. JAK2 Mutations

JAK2 is a member of the family of Janus kinases essential in hematopoiesis. *JAK2*V617F, a mutation in the pseudokinase domain, results in constitutional activation of tyrosine kinase activity. Activation of the JAK/STAT pathways is the hallmark of MPN. *JAK2*V617F mutation is detectable in patients with MDS/MPN, including RARS with thrombocytosis (RARS-T) and a subgroup of patients with del(5q) [11]. *JAK2*V617F-positive MDS has a lower risk of leukemic transformation and better OS [11]. The JAK/STAT pathway is activated in various subtypes of AML. The JAK1/2 inhibitor ruxolitinib is active against secondary AML evolving from MPN [12,69,70]. STAT3 inhibition with tyrosine kinase inhibitors has demonstrated significant anti-proliferative effects in human AML cell lines harboring *JAK2*V617F mutation [69].

### 7.2. FLT3 Mutations

FLT3 is a class III receptor tyrosine kinase consisting of a juxtamembrane domain (JMD), two tyrosine kinase domains (TKD1 and TKD2), and five extracellular immunoglobulin-like domains. It is highly expressed in hematopoietic stem and progenitor cells. Dimerization and auto-phosphorylation follows binding of FLT3 to the FLT3 ligand [71,72,73]. This leads to downstream activation of the PI3K/AKT and RAS/MAPK pathways. *FLT3* ITD occurs in around 30% of AML and is commonly associated with normal karyotype, trisomy 8, t(6;9), and t(15;17) [74,75]. *FLT3*-ITD occurs as a result of an in-frame duplication of 3 to 400 base pairs at the JMD or TKD1 domains. This results in constitutive activation of *FLT3* and aberrant downstream activation of signaling pathways including the STAT5 pathways [76,77]. Clinically *FLT3*-ITD positive AML is associated with higher relapse rates and inferior survivals, which are directly related to the *FLT3*-ITD allele burden [71,78]. Hence, FLT3 inhibition represents an important treatment target. In MDS, *FLT3* mutations occur at a much lower frequency, varying from 0.6% to 6% [15,79,80,81,82,83,84,85]. Previous studies have examined acquisition of *FLT3* mutation in serial samples during disease transformation, showing a higher frequency of *FLT3*-ITD during leukemia transformation (Table 1) [15,81,84,85,86,87,88]. Intriguingly, among patients with MDS transforming into AML with *FLT3*-ITD, more than half had prior exposure to HMAs [88]. These observations suggest that *FLT3* mutations are involved in treatment failure with HMAs and leukemic transformation.

Various FLT3 inhibitors have been tested in prospective clinical trials in *FLT3*-ITD-positive AML. They include midostaurin, tandutinib, KW-2449, lestaurtinib, sunitinib, sorafenib, and quizartinib [71,73,89,90,91,92,93,94,95,96,97,98,99,100,101,102,103,104]. The second generation dual FLT3 and JAK2 inhibitor pacritinib is currently under prospective evaluation [105]. A response rate of 46%–100% can be achieved with the use of FLT3 inhibitors as monotherapy in *FLT3*-ITD+ AML [71]. However, responses were generally brief, with a median duration of remission of only 11–13 weeks [71]. Combination therapy with FLT3 inhibitors and chemotherapy resulted in improved rates of complete remission (CR) or complete response with incomplete hematological recovery (CRi) [71], although this did not translate into an improvement in leukemia-free-survival or OS.

Combination of FLT3 inhibitors with HMAs has also been explored. HMAs have single agent activity in inducing and maintaining remissions in MDS and AML, resulting in the prolongation of OS [106,107,108,109,110,111,112,113]. HMAs increase apoptotic cell death, expression of tumor-necrosis factor (TNF)-related apoptosis inducing ligand (TRAIL), and demethylation of CpG-A elements [114]. HMAs also induce differentiation of the leukemic clones by upregulating differentiation-modulating genes [115,116]. Furthermore, FLT3 ligand is released by the bone marrow microvascular endothelium, T-cells, and myeloid leukemic cells in an autocrine manner [117,118,119]. A surge of FLT3 ligand may occur because of the use of FLT3 inhibitors, resulting in a decrease in their efficacy. The concomitant use of HMAs is postulated to ameliorate this FLT3 ligand surge. Combination of the FLT3 inhibitor midostaurin with decitabine has shown synergism in a phase I clinical study of elderly AML patients [120]. In a prospective phase II trial combining sorafenib and azacitidine, an overall response rate of 46% (CR = 27%, CRi = 16%, partial remission = 3%) and a median response duration of 2.3 months was achieved [117]. The clinical evidence supports the proposition that reduced FLT3 ligand release and surge with HMA results in improved responses to treatment [121,122,123,124]. The addition of azacitidine following sorafenib-induced remission in relapsed or refractory *FLT3*-ITD positive AML improved the duration of remission, successfully bridging patients to subsequent HSCT [16].

Mutations that disrupt the binding of FLT3 inhibitors to FLT3 lead to resistance (at the activation loop, residues D835V/Y/F/H, D839G, Y842C/H; at the gatekeeper residues F691L/I) [71,73,125]. Second generation tyrosine kinase inhibitors (TKI) have been developed to tackle resistance to first generation FLT3 inhibitors. Ponatinib is a multikinase inhibitor with proven efficacy against chronic myelogenous leukemia harboring the *BCR*-*ABL1* T315I mutation [51]. It has demonstrable *in vitro* activities against AML cell lines with *FLT3*-ITD and *FLT3*-TKD mutations, especially N767D, F691, and G697R [126,127,128]. Crenolanib is a novel benzamidine quinolone derivative with highly selective *FLT3* inhibitory activity [129] and *in vitro* efficacy against D835 *FLT3* mutants resistant to sorafenib and quizartinib [129]. Significant clinical activity was seen in patients with relapsed or refractory *FLT3-ITD*+ AML with the *FLT3* D835 mutation [130]. Other targetable non-mutational pathways that cause resistance to *FLT3* inhibitors include the upregulated pro-survival pathways (MEK/ERK, Pi3K/AKT, STAT/PIM), upregulated FLT3 receptor or FLT3 ligand, bone marrow microenvironment/stroma-mediated resistance (CXCL12-CXCR4 pathway), and activated anti-apoptotic proteins [125,131]. With the rapidly developing armamentarium against *FLT3*-mutated AML, targeting *FLT3*-mutated MDS and secondary AML is an attractive therapeutic option.

### 7.3. KIT Mutations

KIT (CD117) is a type III tyrosine kinase receptor [132]. *KIT* mutations in exon 8 result in activation of the PI3K/Akt pathways, and those in codon 816 (activation loop) result in STAT3/STAT5 and PI3K/Akt pathway activation. *KIT* mutations are detected in 20%–30% of patients with core-binding factor (CBF) leukemias that involve t(8;21) and inv(16)/t(16;16). *KIT* mutations in CBF leukemias are associated with shorter survivals [133]. The combination of the multikinase inhibitor imatinib with chemotherapy was active against AML harboring *KIT* exon 8 mutations. Phase I/II trials of dasatinib (active against the *KIT* codon 816 mutation) in combination with induction chemotherapy in CBF AML are ongoing [132]. *KIT* mutations are found in 1% of MDS, but clinical benefits of KIT inhibition in such patients remain to be defined.

### 7.4. CBL Mutations

*CBL* is a tumor suppressor gene that negatively regulates receptor tyrosine kinases [11]. *CBL* mutations are observed in about 1% of MDS, but with higher frequencies of 19% in CMML and 10% in MDS/MPN. Mutant CBL is unable to attenuate signaling functions that drive oncogenesis. It has been shown that, in juvenile myelomonocytic leukemia, mutant CBL confers resistance to apoptosis via activation of the Src family of kinases and thus the lyn-PI3K/AKT pathway [11]. Lyn-PI3K/AKT is a potentially targetable pathway in *CBL*-mutated myeloid malignancies [12]. *CBL* mutations are associated with high-grade MDS [134,135,136].

### 7.5. Mutations in the RAS Pathway

Important members of the RAS family include N-RAS, K-RAS and H-RAS. They are GTPases that regulate cell proliferation, differentiation, and survival [11]. *N-RAS* mutations are seen in 4%–9% of MDS and 12% of CMML [134]. *K-RAS* mutations are more commonly seen in up to 14% of CMML [134]. The prognostic impact of *N-RAS* and *K-RAS* mutations is yet to be determined. Other members of the RAS pathway include *NF-1*, *BRAF*, and *PTPN-11*, but mutations involving these gene are rarely seen in patients with MDS [12].

### 7.6. Mutations in the Cohesin Gene Family

The cohesin complex is responsible for the cohesion of sister chromatids, DNA repair, and regulation of transcription. Cohesin complex gene mutations or deletions that are seen in myeloid malignancies include *STAG2*, *RAD21*, *SMC1A*, and *SMC3* [11]. They are mutually exclusive. *STAG2* mutations are seen in approximately 10% of MDS and are associated with *RUNX1* mutations commonly seen in high-grade MDS [11]. The impact of cohesin complex gene aberrations on the outcome of MDS is not well defined [11].

## 8. Other Novel Targeted Approaches

### 8.1. BCL-2 Inhibition

The intrinsic cellular apoptotic pathway is controlled by the BCL-2 family of proteins comprising pro-apoptotic and anti-apoptotic proteins [137,138]. Important pro-apoptotic proteins include the BAX/BAX-like proteins and the BH3-only proteins. They cause cellular apoptosis via activation of caspase-3 and casepase-7 [138]. The anti-apoptotic or “pro-survival” BCL-2 family members include BCL-2, MCL-1 or BCL-X_L_. Aberrant apoptosis is a cardinal feature of MDS owing to deregulated balance between pro-apoptotic and anti-apoptotic BCL-2 proteins [138]. In low-risk MDS, intramedullary apoptosis is increased. In high-risk MDS and secondary AML, there is an acquired resistance to apoptosis associated with a balance shift towards an increased expression of anti-apoptotic BCL-2 proteins [138]. The aberrant apoptotic pathway in MDS is a potential target. It has been shown that inhibition of the anti-apoptotic proteins by ABT-737 or ABT-199 targeted hematopoietic stem and progenitor cells in high-risk MDS and secondary AML samples [137,138,139,140]. The anti-apoptotic BCL-2 family of proteins modulates the anti-leukemic effects of azacitidine, and profiling of these proteins might predict response to azacitidine [137,141,142]. Therefore, targeting of the BCL-2 family of proteins may enhance azacitidine effectiveness, so that a combination of BCL-2 inhibitors with HMAs is an attractive option, particularly in cases of failing azacitidine treatment.

### 8.2. Polo-Like Kinases

Polo-like kinases (Plks) are serine/threonine kinases that regulate cell cycle, including entry into mitosis, DNA replication, and DNA damage responses [143]. Over-expressed in AML, Plk1 is of particular importance, playing a critical role in mitosis, protection against apoptosis, and cancer invasiveness. Volasertib, a dihydropteridinone derivative, is a small molecule competitive kinase inhibitor of Plk1. It is active against relapsed or refractory AML, either as monotherapy or in combination with low-dose cytarabine [143].

### 8.3. Mitogen-Activated Protein Kinase (MAPK) and Mammalian Target of Rapamycin (mTOR)

The mitogen-activated protein kinase (MAPK) and the mammalian target of rapamycin (mTOR) pathways are involved in the expansion and survival of leukemic cells [69]. The downstream effectors of MAPK, namely Mnk kinases, are potential targets for treatment in AML. Inhibition of mTOR complex 1 and mTOR complex 2 has shown potent anti-leukemic effect [69].

### 8.4. Arsenic Trioxide

Arsenic trioxide (As_2_O_3_) has been shown to be active against MDS via its pro-apoptotic, anti-proliferative, and anti-angiogenic properties [144]. As_2_O_3_ promote apoptosis via downregulation of the BCL-2 family of proteins, inhibition of glutathione peroxidase activity, caspase activation, and NF-κB inhibition [144,145,146]. As_2_O_3_ as a single agent or in combination results in a response in about 25% of cases [144,147,148]. Combined use with thalidomide, gemtuzumab ozogamacin (GO), and azacitidine has been reported [149,150,151]. The combination of arsenic trioxide with thalidomide has shown activity against MDS with inv(3)(3q21;3q26), targeting the EVI1 pathway [144].

### 8.5. Monoclonal Antibodies and Novel Immunotherapy

Gemotuzumab ozogamacin (GO) is an immuno-conjugate composed of the anti-CD33 monoclonal IgG4 antibody conjugated to the cytotoxin calicheamicin [27,69,152]. There is clinical benefit, which has been shown recently, in the management in older AML patients [69]. SGN-CD33a is an anti-CD33 monoclonal antibody conjugated to pyrrolobenzodiazepine dimer [69]. It cross-links DNA leading to cell death and is as least three times as potent as GO in preliminary studies. CD123 (IL-3R) is a leukemia stem cell marker targetable using immuno-conjugates, bi-specific antibodies (combined with diphtheria toxin and an antibody against CD95), and engineered T-cells expressing chimeric antigen receptors (CAR-T) [27,69]. Deregulation of the programmed cell death (PD)-1/PD-L1 axis has also been shown to contribute to the pathogenesis of myeloid malignancies including AML and MDS [153]. PD-L1 is over-expressed in various malignancies. Blockade of the PD-1 pathway in solid tumors results in CD8+ T-cell infiltration, leading to clinical response [69]. Phase I studies of immune checkpoint inhibitors in acute myeloid leukemia and myelodysplastic syndromes are now ongoing.

## 9. Conclusions and Future Directions

Data arising from whole-genome sequencing (WGS) have shown that the clonal evolution of MDS to AML is complex [154]. More tantalizing is the observation that, in individuals who are either healthy or have minor cytopenias without definite evidence of MDS, next-generation sequencing has shown the presence of MDS-related mutations in genes including *ASXL1*, *TP53*, *BCORL1*, *GNAS*, *SF3B1*, *DNMT3A*, *TET2*, and *JAK2*. The frequency of detecting these mutations increases with age, being very rare in people <40 years old, but present in about 10% of people aged 70 to 79 years, and reaching 18% in people >90 years old. The risk of development of MDS or leukemia in these people has been estimated to be 0.5%–1% per year. This condition, referred to as clonal hematopoiesis of indeterminate potential (CHIP), highlights the dynamic nature of clonal evolution in myeloid cells [155]. When MDS has arisen, the selection of clones during transformation is shaped by acquisition of genetic alterations during clonal expansion, as well as exposure to genotoxic chemotherapy [156].

Better understanding of the molecular landscape of MDS has important clinical implications. Firstly, prognosticating MDS based on molecular aberrations will supplement current models in stratifying patients for treatment including allogeneic HSCT. Somatic mutations of *TP53*, *TET2*, and *DNMT3A* have been shown to identify patients with MDS with shorter OS after allogeneic HSCT [157], suggesting that newer treatment strategies other than transplantation are needed for these patients. Secondly, molecular markers may better predict response and resistance to treatment with HMAs. Thirdly, detection of targetable molecular markers during treatment resistance or leukemic transformation may provide an opportunity for specific therapy, as exemplified by the use of FLT3 inhibitors in *FLT3*-ITD positive secondary AML. Table 2 summarizes the molecular aberrations in MDS with respect to their prognostic impact and their targetability. Finally, understanding the molecular dynamism of myeloid cell mutation has important implications on treatment, particularly in detecting CHIP that precedes the development of clinical MDS [155,156]. Currently, there is as yet no specific treatment for these mutations. However, rapid advances in molecular biology and drug development lead to the optimism that these mutations may soon be targetable, with a view to early treatment perhaps even at the stage of CHIP so as to avert the development of myeloid malignancies. Hence, future treatment strategies for MDS may involve exploitation of genetic information in designing more effective therapy encompassing single agents or combinatorial approaches.

## Figures and Tables

**Table 1:** Previous studies evaluating *FLT3*-ITD mutations in patients with MDS during disease progression.

Study	Patient No.	Treatment	*FLT3* Mutations at Dx	sAML No.	*FLT3* Mutations at sAML	HMA Use in Acquired *FLT3* Mutation
Shih *et al.* [86]	70 *	Supportive; Ara-C; oral chemotherapy	ITD: 3 (4.3%)	70 *	ITD: 10 (14.2%)	No
			TKD: 1 (1.4%)		TKD: 4 (5.7%)	
Georgiou *et al.* [87]	97	Not reported	ITD: 1 (1%)	42	ITD: 5 /42 (12%)	Not reported
			TKD 1 (1%)		TKD: 1/42 (2.4%)	
			ITD + FLT-TKD: 1 (1%)		ITD + TKD: 1/42 (2.4%)	
Georgiou *et al.* [81]	50	Not reported	ITD: 0	2	ITD: 2 #	Not reported
Dicker *et al.* [84]	20 **	Supportive care	ITD: 0	20 **	ITD: 4 (20%)	No
Takahashi *et al.* [88]	278	Supportive/HMA/induction chemotherapy/HSCT	ITD: 2	74	ITD: 11 (15%)	ITD: 6/11 (54.5%)
			TKD: 2		TKD: 4 (5.4%)	TKD: 2/4 (50%)
Badar *et al.* [85]	102	HMA: 75 (73%)	ITD: 0	27	ITD: 5 (19%)	Not reported
		Immunomodulators: 7 (7%)	TKD: 0			
		Growth factors: 4 (4%)				
		HSCT: 10 (10%)				
		Others: 16 (16%)				
Meggendorfer *et al.* [15]	38	Not reported	ITD: 0	38	ITD: 6 (16%)	Not reported
			TKD: 0		TKD: 3 (8%)	

MDS: myelodysplastic syndrome; No.: number; Dx: diagnosis; sAML: secondary acute myeloid leukemia; HMA: hypomethylating agent; ITD: internal tandem duplication; TKD: tyrosine kinase domain mutation; *: 70 patients with sAML were retrospectively evaluated for *FLT3* mutation at sAML and MDS stages; #: *FLT3*-ITD was evaluated serially at 6 and 12 months in this study. *FLT3*-ITD was detected at 6 months in 2 cases while still at MDS stage. Both progressed to sAML; **: 20 paired samples at MDS and sAML were evaluated; HSCT: hematopoietic stem cell transplantation.

**Table 2:** Recurrent mutations in myelodysplastic syndrome with prognostic and treatment implications.

Mutated Genes	Function	Frequency	Prognostic Impact	Potential Targetted Therapy	References
**DNA methylation**					
*TET2*	Conversion of 5mC to 5-hmC	20%–30% in MDS 50%–60% in CMML	None	DNA methyltransferase inhibitor (DNMTI)	[1,11,17,18,19,20,21,22,23,24,25]
*DNMT3A*	DNA methyltransferase, histone methylation and transcription repression	10% in MDS	Unfavorable	DNMT1	[26,27,28,29,32,33,34]
*IDH1/IDH2*	Convert isocitrate to α-KG, regulates TET2	5%	Unfavorable	DNMT1, IDH1/2 inhibitors	[27,28,35,36,37,38,39,40,41]
**Histone modification**					
*EZH2*	Histone methyltransferase, gene repression	5%	Unfavorable	HDAC inhibitors, EZH2 inhibitors	[28]
*ASXL1*	H3 methylation	15%–20%	Unfavorable	HDAC inhibitors	[11,28,42,43,44,45]
*UTX*	H3K27 demethylase	1%	None	HDAC inhibitors	[11,28,46,47]
**RNA splicing**					
*SF3B1*	Pre-mRNA splicing	15%–30%	Favorable	None	[11,19,49,50]
*SRSF2*	Spliceosome assembly	10%–20%	Unfavorable	None	[11,51,52,53]
*ZRSR2*	Spliceosome assembly	<5%	None	None	[54]
*U2AF1*	Spliceosome assembly	5%–10%	None	None	[11,54]
**Transcription**					
*RUNX1*	Regulates myeloid differentiation	10%	Unfavorable	None	[11,55,56,57]
*BCOR/BCORL1*	BCL6 co-repressor	5%	Unfavorable	HDAC inhibitors	[58,59,60,61]
*ETV6*	ETS transcription factor	<5%	Unfavorable	None	[11,62]
*SETBP1*	Cell proliferation	2%–5%	Unfavorable	None	[11,63,64]
*GATA2*	Transcriptional activator	Rare	Unfavorable	None	[11]
**DNA repair**					
*TP53*	Cell cycle regulation, tumor suppressor gene	10%	Unfavorable	Antisense TP53 oligonucleotide	[11,65,66,67,68]
**Signal transduction**					
*JAK2*	Tyrosine kinase activation	5%	None	JAK1/2 inhibitors	[11,69,70]
*FLT3*	Tyrosine kinase activation	<5%	Unfavorable	FLT3 inhibitors	[15,16,71,81,82,83,84,85,86,87,88,89,90,91,92,93,94,95,96,97,98,99,100,101,102,103,104,105]
*KIT*	Tyrosine kinase activation	Rare	None	TKI (imatinib, dasatinib)	[132,133]
*CBL*	Proto-oncogene	5%	Unfavorable	None	[11,12,134,135,136]
*RAS* pathway	GTPase signal transduction	10%	Unfavorable	None	
**Cohesin complex**					
*STAG2*	Control of cell division	5%–10%	Unfavorable	None	[11]
*RAD21*	Component of cohesin complex	<3%	None	None	[11]
*SMC3*	Structure and function role in cohesin complex	<3%	None	None	[11]

MDS: myelodysplastic syndrome; CMML: chronic myelomonocytic leukemia; α-KG: α ketoglutarate; HDAC: histone deacetylase; TKI: tyrosine kinase inhibitor.
